# Representation of Spatial and Feature Information in the Monkey Dorsal and Ventral Prefrontal Cortex

**DOI:** 10.3389/fnint.2018.00031

**Published:** 2018-08-07

**Authors:** Christos Constantinidis, Xue-Lian Qi

**Affiliations:** Department of Neurobiology and Anatomy, Wake Forest School of Medicine, Winston-Salem, NC, United States

**Keywords:** prefrontal cortex, monkey, neurophysiology, fMRI, neurons

## Abstract

The primate prefrontal cortex (PFC) is critical for executive functions including working memory, task switching and response selection. The functional organization of this area has been a matter of debate over a period of decades. Early models proposed segregation of spatial and object information represented in working memory in the dorsal and ventral PFC, respectively. Other models emphasized the integrative ability of the entire PFC depending on task demands, not necessarily tied to working memory. An anterior-posterior hierarchy of specialization has also been speculated, in which progressively more abstract information is represented more anteriorly. Here we revisit this debate, updating these arguments in light of recent evidence in non-human primate neurophysiology studies. We show that spatial selectivity is predominantly represented in the posterior aspect of the dorsal PFC, regardless of training history and task performed. Objects of different features excite both dorsal and ventral prefrontal neurons, however neurons highly specialized for feature information are located predominantly in the posterior aspect of the ventral PFC. In accordance with neuronal selectivity, spatial working memory is primarily impaired by inactivation or lesion of the dorsal PFC and object working memory by ventral inactivation or lesion. Neuronal responses are plastic depending on task training but training too has dissociable effects on ventral and dorsal PFC, with the latter appearing to be more plastic. Despite the absence of an overall topography, evidence exists for the orderly localization of stimulus information at a sub-millimeter scale, within the dimensions of a cortical column. Unresolved questions remain, regarding the existence or not of a functional map at the areal and columnar scale, and the link between behavior and neuronal activity for different prefrontal subdivisions.

## Introduction

The functional organization of the prefrontal cortex (PFC) in humans and non-human primates has been a matter of long-standing debate (Riley and Constantinidis, 2016). Neurophysiological studies in non-human primates in the 1990s described physiological correlates of functional specialization across the dorsal-ventral axis, proposing that the dorsolateral PFC is responsible for spatial working memory, and the ventrolateral PFC for object working memory (Wilson et al., 1993; Ó Scalaidhe et al., 1997, 1999). These conclusions were challenged by other influential studies, which demonstrated that individual neurons in the PFC can represent both spatial and object information, thus suggesting that PFC is the site of integration of these streams in the primate brain (Rao et al., 1997; Rainer et al., 1998a). Human imaging studies at the time were also equivocal about the dorso-ventral organization of the PFC, with some supporting the idea that specialized processing occurs within the two prefrontal subdivisions (Adcock et al., 2000; Leung et al., 2002; Sala and Courtney, 2007), whereas others suggesting an organization in terms of cognitive operations rather than type of information (Owen et al., 1996, 1998; Stern et al., 2000). An anterior-posterior axis of functional specialization has also been speculated, suggesting a hierarchical organization progressing towards anterior areas (Badre and D’Esposito, 2009). How such a hierarchy might intersect with a dorso-ventral specialization added to the confusion.

A number of recent studies allow a re-evaluation of this debate, resolution of some of the questions and deeper insights on the representation of information in prefrontal networks. Here, we review results from non-human primate neurophysiology. We discuss the anatomical organization of the PFC, the evidence for specialization of stimulus representation within the PFC, the capacity for plasticity based on task being executed, the evidence for functional specialization drawn from inactivation experiments, and finally, the evidence for orderly representation of stimulus information within the cortical microcircuits.

## Anatomical Organization

The PFC can be divided into a lateral, a medial and an orbital aspect. The lateral aspect can be further distinguished into a dorsal and ventral subdivision. The terms ventral and dorsal have not been used consistently in the literature, however (Constantinidis and Procyk, 2004). Walker initially identified multiple cytoarchitectonic areas on the lateral aspect of the macaque brain: areas 8a (encompassing the Frontal Eye Field) and 45 lining the superior and inferior banks of the arcuate sulcus respectively, area 8b just medial to the arcuate, areas 9 and 12 in the superior and inferior convexities of the cortex respectively, area 46 lining either banks of the principal sulcus and area 10 covering the frontal pole (Walker, 1940). Area 46 has been additionally subdivided along its mediolateral aspect, into areas 46dr, 46d, 46v and 46vr, lining the medial rim, the medial and the lateral banks of the principal sulcus and the lateral rim of the principal sulcus, respectively (Preuss and Goldman-Rakic, 1991). There is also evidence of a specialization in the anterior-posterior aspect, with the caudal aspect of area 46 shown to be functionally dissociable from the anterior one; the former is sometimes referred to as area 9/46, whereas the most anterior one as area 46 (Petrides, 2000). More areas yet may be present based on the evidence provided by fMRI studies probing functional connectivity at rest (Goulas et al., 2017).

Anatomical studies point to a relative segregation of projections from the posterior parietal cortex, which terminate mostly to the dorsal PFC (areas 8 and 46, including both banks of the principal sulcus), and from the inferior temporal cortex, which terminate on areas 12 and 45 of the ventral PFC (Petrides and Pandya, 1984; Selemon and Goldman-Rakic, 1988; Cavada and Goldman-Rakic, 1989). This specificity of inputs is not only limited to visual afferents; auditory connections representing sound localization information also target the dorsal PFC, whereas those representing auditory features terminate in the ventral PFC (Romanski et al., 1999). A relative segregation of inputs has also been observed across the anterior-posterior axis, with areas higher in the sensory and limbic hierarchies projecting to more anterior prefrontal subdivisions (Gerbella et al., 2013; Barbas, 2015; Borra et al., 2017).

**Figure 1:**
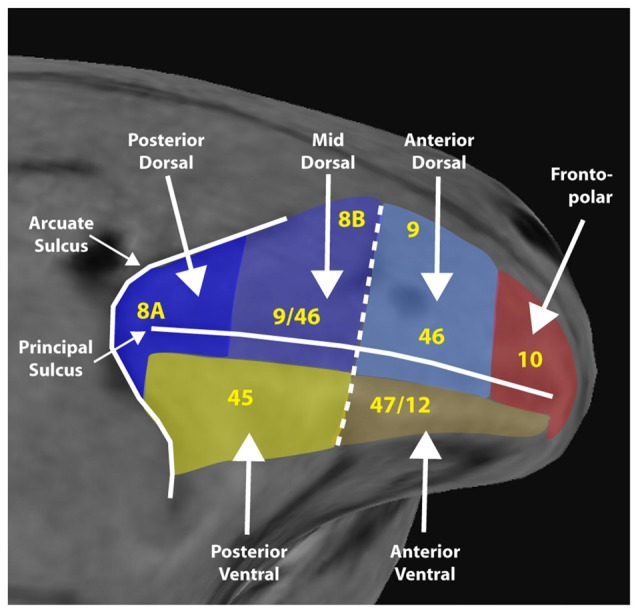
Diagram of a macaque monkey brain, with the lateral prefrontal cortex (PFC) divided into six regions, as indicated. Yellow letters denote approximate location anatomical areas, based on the Petrides and Pandya nomenclature. Adapted from Riley et al. (2017); with permission.

Based on anatomical and physiological evidence, we have recently proposed a functional division of the lateral PFC into a dorsolateral region that encompasses the area between the arcuate and principal sulcus and both banks of the principal sulcus, and a ventral region that comprises the inferior limb of the arcuate and the inferior convexity (Riley et al., 2017). These are further subdivided along the anterior/posterior axis. A fronto-polar aspect completes the lateral surface (Figure 1).

## Localization of Stimulus Selectivity

As noted in the “Introduction” section, neurophysiological studies initially proposed that dorsolateral PFC subserves primarily spatial working memory whereas ventrolateral, object working memory (Wilson et al., 1993; Ó Scalaidhe et al., 1997, 1999). This conclusion was based on the finding that a greater proportion of neurons selective for the spatial location of peripheral stimuli were observed in the dorsolateral than in the ventrolateral PFC, in monkeys trained to perform spatial working memory tasks (Wilson et al., 1993). On the other hand, neurons selective for highly specialized images such as faces were almost exclusively observed in the ventrolateral PFC, at least in monkeys just required to view such stimuli passively (Ó Scalaidhe et al., 1997, 1999). This “domain-specific” organization can be thought of as an extension of the dorsal and ventral visual streams (Ungerleider and Mishkin, 1982; Felleman and Van Essen, 1991).

An opposing view posited that prefrontal neurons selective for both the location of stimuli and their identity can be encountered throughout the PFC, after monkeys have been trained in tasks that required them to remember both the location and identity of a stimulus (Rao et al., 1997; Rainer et al., 1998a). The implication drawn was that the functional specialization observed in the earlier studies was the result of task requirements (Rao et al., 1997; Rainer et al., 1998a). This “integrative” model suggested a plastic prefrontal organization that is shaped by cognitive demands imposed by the task, instead. Related models suggest that the PFC is primarily organized based on cognitive process rather than stimulus presentation (Owen et al., 1998). Some studies have also suggested that lesions of the ventral PFC do not impair working memory for stimulus shape and color (Rushworth et al., 1997).

### Spatial Selectivity

A number of recent studies in our own and other laboratories have reexamined the selectivity of PFC for different properties of stimuli by comparing responses of dorsal and ventral prefrontal neurons to the same stimuli, in the same animals. Spatial selectivity proved a strong predictor of whether a neuron was recorded in the dorsal or the ventral PFC (Meyer et al., 2011). More neurons in the dorsolateral PFC responded to white square stimuli presented at varying spatial locations; a higher percentage of these neurons were selective for spatial location; and a greater spatial selectivity was observed among those neurons, compared to ventrolateral prefrontal neurons. This difference in spatial selectivity between dorsal and ventral PFC was true for monkeys naïve to any training that viewed stimuli presented at different locations passively; for monkeys trained to perform only spatial working memory tasks; as well as for monkeys that were explicitly trained to perform tasks that required simultaneous maintenance of spatial and object information in memory (Meyer et al., 2007, 2011; Riley et al., 2017). This is not to say that ventral prefrontal neurons exhibit no selectivity for spatial location. Neurons with well localized receptive fields and significant selectivity for spatial location were observed in the ventral PFC (Meyer et al., 2011; Riley et al., 2017).

**Figure 2:**
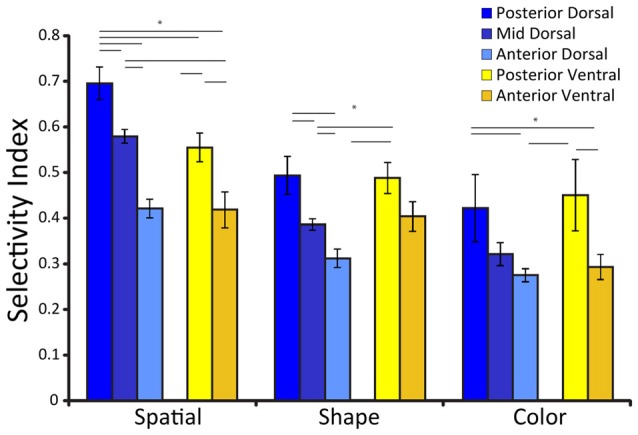
Average selectivity index for three different stimulus sets (defined as the difference between the maximum and minimum firing rate for the stimuli of the set, divided by their sum), among neurons with significant responses to stimuli for each prefrontal subdivisions. Error bars represent standard error of the mean. Adapted from Riley et al. (2017), with permission. Horizontal lines with stars * indicate significant differences (*p* < 0.05) between conditions, determined based on a 2-way ANOVA and *post hoc* Tukey test.

Spatial selectivity is dependent on the position of neurons along the anterior-posterior axis (Figure 2). Neuronal selectivity was found to decrease along the anterior posterior axis, so that the most highly selective neurons for stimulus properties were located more posteriorly in the PFC (Riley et al., 2017). Conversely, neurons in more anterior areas exhibited little selectivity to stimuli *per se* but were more likely to represent task variables. Thus, the functional specialization between dorsal and ventral PFC became less meaningful, particularly as task demands molded responses of neurons to different stimuli.

Stimulus selectivity also appears to have a strong temporal component in the PFC. In our experiments, neurons were highly selective for spatial location early in the trial, and response latency itself differentiated the highly selective posterior areas from the less selective anterior areas (Riley et al., 2017). Other studies too have determined a stronger contralateral bias in neuronal responses shortly after the appearance of the stimulus, which dissipated later in time (Kadohisa et al., 2015).

Finally, spatial selectivity was most evident for peripheral stimuli. In our experiments, stimuli were positioned 10–14° away from the fovea. Studies that have tested stimuli within 4–6° degrees from the fovea have found little or no differentiation between dorsal and ventral PFC (Rao et al., 1997; Kadohisa et al., 2015). We note that properties of ventral prefrontal neurons are similar to inferior temporal neurons, which project to the ventral PFC, and which can be highly selective for spatial position, particularly in perifoveal locations (DiCarlo and Maunsell, 2003). The difference in spatial selectivity between dorsal and ventral PFC is therefore quantitative rather than qualitative, yet clearly distinguishing between the two.

### Feature Selectivity

Our experiments probed further the selectivity of prefrontal neurons for object features by testing neuronal responses in the two areas with a set of eight white geometric objects. Feature selectivity provided a less straightforward picture of the two prefrontal subdivisions. A larger proportion of dorsolateral prefrontal neurons responded to any stimulus appearing in their receptive fields. Many of these neurons exhibited broad but significant selectivity for shape (Meyer et al., 2011). These results are in agreement with findings from other laboratories, which have found broad but significant selectivity for stimulus shape in the dorsolateral PFC, including the Frontal Eye Fields (Peng et al., 2008; Clark et al., 2012). Such shape selectivity has also been reported in the posterior parietal cortex (the main afferent input to dorsolateral PFC), where broad but significant tuning for stimulus shape is present, when probed with very similar geometric shapes (Sereno and Maunsell, 1998; Janssen et al., 2008). On the other hand, ventrolateral prefrontal neurons were less likely to be driven by any stimulus, at least among the limited set of geometric shapes. When we quantified shape selectivity among responsive neurons, this was higher in the ventrolateral than dorsolateral PFC prior to training (Meyer et al., 2011). However, the percentages of selective neurons and selectivity magnitude values after training in working memory tasks were not found to be significantly different between the two prefrontal subdivisions (Meyer et al., 2011). This result essentially replicated the findings of the earlier studies that failed to detect a difference in feature selectivity between dorsal and ventral prefrontal neurons, in monkeys that were trained to perform a combined spatial and object working memory task (Rao et al., 1997). Newer studies have also found little evidence for greater prevalence of object coding in ventral compared to dorsal PFC, when each neuron is tested with a few stimuli (Kadohisa et al., 2015).

Although a limited stimulus set does not allow detection of a clear-cut dichotomy in shape selectivity between dorsal and ventral PFC, the lack of overall responsiveness in the ventrolateral PFC is not incompatible with high specialization for stimulus features. Neurons that are highly selective for object features, will only respond vigorously to a limited set of stimuli and are likely to produce uniformly weak responses to stimuli drawn from a small set, failing to differentiate between them. Such a response pattern has precisely been reported for inferior temporal neurons (Gross et al., 1972; Desimone et al., 1984; Tanaka et al., 1991; Fujita et al., 1992). In contrast, when neurons were tested with stimulus sets requiring very narrow shape selectivity, such as faces and complex objects, neurons distinguishing between such stimuli were localized exclusively in the ventral PFC (Ó Scalaidhe et al., 1997, 1999).

Our experiments also evaluated functional specialization for color, relying on eight iso-luminant colored squares presented over the fovea. The results of this analysis mirrored that of feature selectivity. A large proportion of dorsal PFC responded to colored squares, with a small percentage of neurons exhibiting weak but significant selectivity (Riley et al., 2017). Other studies have also observed color selectivity in only a small proportion of prefrontal neurons, in the order of 5%–15% (Lara and Wallis, 2014). Similarly, approximately 15% of posterior parietal neurons show selectivity for color of stimuli (Constantinidis and Steinmetz, 2001). A smaller percentage of ventral prefrontal neurons responded to the colored squares, and their overall selectivity for color was not significantly different from that of the dorsal PFC (Riley et al., 2017). Combined fMRI studies and neurophysiological studies in the temporal lobe have suggested that neurons selective for faces, other shapes, and colors are clustered at distinct patches of cortex (Tsao et al., 2006; Popivanov et al., 2014; Chang et al., 2017). A handful of studies have explored the PFC as well, for example suggesting that color-selective neurons are concentrated in specific “patches” (Lafer-Sousa and Conway, 2013). These results argue strongly for precise localization of function within the PFC.

As was the case for spatial stimuli, feature selectivity depended upon a second anatomical dimension, the position of neurons along the anterior-posterior axis (Figure 2). Neuronal selectivity for shape and color too was found to decrease along the anterior posterior axis, so that the most highly selective neurons for stimulus properties were located more posteriorly in the PFC (Riley et al., 2017). Object representation is also time dependent; early responses represent objects, whereas more abstract information such as stimulus category has been documented later in prefrontal responses (Meyers et al., 2008; Kadohisa et al., 2015).

### Plasticity of Stimulus Representations

The activity of prefrontal neurons is well known to be modulated by task demands. Neuronal activity has been shown to represent the abstract rules of the cognitive tasks subjects are trained to perform (White and Wise, 1999; Wallis et al., 2001), as well as categories (Freedman et al., 2001; Shima et al., 2007), and numerical quantities (Nieder et al., 2002). Responses to stimuli are also modulated by perceptual decisions (Kim and Shadlen, 1999; Barraclough et al., 2004), reward expectation (Leon and Shadlen, 1999), and sequences of events or actions (Averbeck et al., 2002; Inoue and Mikami, 2006; Sigala et al., 2008; Berdyyeva and Olson, 2010). Activity of single neurons can represent stimulus features and task variables simultaneously, including nonlinear combinations of these factors (Rigotti et al., 2013; Parthasarathy et al., 2017). Additionally, prefrontal neurons respond more strongly to stimuli when these are part of a task and the monkey is required to attend to them, than the same stimuli when they are not necessary for performing the task or are even distracting in the context of the task (Rainer et al., 1998b; Everling et al., 2002; Lennert and Martinez-Trujillo, 2011). The stimulus dimension being represented in neuronal activity may dynamically change during the course of a single trial (Mante et al., 2013). The representation of task variables may reasonably be assumed to be the result of training, as these differ between trained tasks. Considering such plasticity of neuronal representations exists, an extreme view of plasticity may lead to the conclusion that all stimulus properties represented in neuronal responses are the effect of training rather than inherent specialization for different types of stimuli. It is important therefore to consider how training in a behavioral task alters stimulus representations within the PFC. Experiments in our laboratory directly addressed this question by recording neuronal activity in monkeys when they were naïve to cognitive task training, and after they were trained to perform tasks that required them to maintain the properties of the same stimuli in working memory (Qi et al., 2011; Qi and Constantinidis, 2013). The effects of training varied between areas. Posterior dorsal PFC exhibited the weakest effects of plasticity, with robust selectivity for spatial stimuli being present both before and after training in the working memory tasks (Meyer et al., 2011; Riley et al., 2017). On the other hand, the ventral subdivisions of the PFC in general, were more affected by the effects of training, such that more neurons were responsive to stimuli when the monkey was performing a working memory task (Meyer et al., 2011; Riley et al., 2017).

Considering this difference in plasticity between ventral and dorsal PFC, is it possible to entirely reverse the relative selectivity for spatial information between the two subdivisions, and have ventral PFC exhibit strong spatial selectivity if this is relevant for a trained task and tightly coupled to reward? Results from one series of studies demonstrated such a reversal; a systematic difference in spatial selectivity between dorsal and ventral PFC was found, but higher spatial selectivity was present in the ventral rather than the dorsal PFC (Kennerley and Wallis, 2009). Recordings in these experiments were localized in the anterior PFC, using a task which signaled the magnitude of reward. It is likely that neuronal responses in anterior and ventral areas are more sensitive to task variables and cognitive factors rather than stimulus properties *per se*, so that robust selectivity to the location of stimuli may emerge as a result of training in task that requires tracking of reward. Ventral PFC has greater sensitivity to learning of new, rewarded conditions likely due to the action of dopamine D1R receptors (Puig and Miller, 2012).

Our experiments also tested the idea that task demands that require memory of *both* location and identity of an object modifies the properties of neuronal firing so as to exhibit selectivity for both spatial and object information, as was speculated in early studies that reported integration of these types of information (Rao et al., 1997). This was clearly not the case. Neurons with selectivity for both identity and location were present prior to training, in both the dorsal and ventral PFC (Meyer et al., 2011). Among neurons responding to stimuli, the average strength of selectivity for either spatial location or shape actually *declined* after training in the task that required working memory for both (Meyer et al., 2011). However, since more neurons were active, the information about stimulus location and shape that could be decoded from neuronal populations after training were no less than that before training (Meyers et al., 2012).

**Figure 3:**
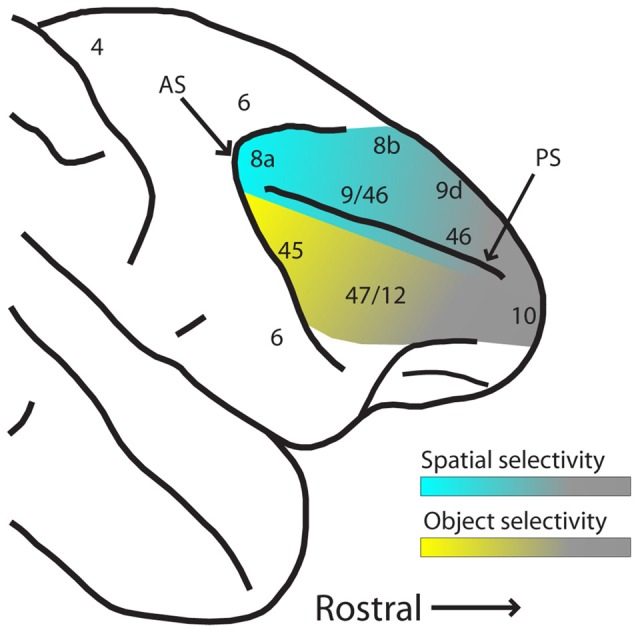
Schematic diagram of hypothesized spatial and object selectivity gradients in the dorsal and ventral PFC. The posterior aspects of dorsal and ventral PFC are most selective for spatial locations and object features (indicated by blue and yellow hues, respectively). Selectivity declines more anteriorly (indicated by gray hue), and effects of task variables and training history become more dominant. Abbreviations: AS, arcuate sulcus; PS, principal sulcus.

These results suggest that while PFC has a great capacity for plasticity and representation of abstract relationships for behaviorally relevant stimuli, plasticity does not entirely erase pre-existing functional specialization for spatial and object information between dorsal and ventral PFC, particularly in their posterior aspects. The gradients of spatial and object selectivity are depicted schematically in Figure 3. Training does not affect all areas of the PFC in the same manner, either. Neuronal properties detected in trained monkeys thus depend on the anatomical location of the neurons.

## Functional Implications of Dorsal and Ventral Prefrontal Inactivation

Although selectivity to different types of stimuli is revealing, the ultimate functional role of an area can be probed by examining the consequences of its activity on performance of working memory tasks. Thus, experiments manipulating neuronal activity can be very informative on the functional roles of prefrontal subdivisions. Temporary inactivation experiments e.g. injections of the GABA_A_ agonist muscimol, or lidocaine, or by cooling of the underlying cortex during working memory are consistent with the specialized stimulus selectivity of dorsal and ventral PFC neurons. Inactivation of dorsal prefrontal areas, including the Frontal Eye Field, decreases performance during spatial working memory tasks (Sommer and Tehovnik, 1997; Dias and Segraves, 1999; Chafee and Goldman-Rakic, 2000; Suzuki and Gottlieb, 2013; Noudoost et al., 2014). Similarly, limited injections of muscimol in the dorsal PFC produce spatial working memory deficits that localize in the contralateral visual field as do small, focal lesions, a phenomenon that has been termed a “mnemonic scotoma” (Funahashi et al., 1993; Sawaguchi and Iba, 2001).

Inactivation of a dorsal area, the Frontal Eye Field, has negligible effects on object working memory (Clark et al., 2014), even when monkeys are tested with objects for which Frontal Eye Field neurons were shown to exhibit broad but significant selectivity in the same object-working memory task (Clark et al., 2012). In contrast, inactivation of the ventral PFC impairs the ability to locate objects based on remembered features, but not on spatial location (Bichot et al., 2015). Location along the anterior-posterior axis is also critical for the effects of lesions. It was lesions in the anterior aspect of the ventral PFC (area 47/12) that failed to produce deficits of feature working memory (Rushworth et al., 1997), and inactivation of the posterior ventral PFC that did (Bichot et al., 2015).

Although our review focuses on spatial and object working memory, we do not wish to suggest that these are the only functions of the dorsal and ventral PFC, respectively. The representation of task rules and associations reviewed in the neurophysiological studies of the previous section fares prominently on the functional consequences of lesion studies. In this case too, the effects of lesions are dissociable between dorsal and ventral PFC. Lesions in the posterior-dorsal PFC impair tasks relying on learnt associations (Petrides, 2005). In contrast, lesions of the mid-dorsal PFC result in deficits in tasks involving presentation of a stimulus and after a delay period, the same stimulus plus a new one, requiring selection of the newly added item, particularly if this is not a novel object but it is one the monkey is familiar with (Petrides, 2005). Damage to the ventral PFC does not produce impairments in recognition or simple recall; its effects become apparent in free-recall tasks (Petrides, 1996). The ventral PFC also appears to be essential for learning a task by trial and error, and reversal learning, which requires learning new associations within a session (Rushworth et al., 1997; Buckley et al., 2009; Rygula et al., 2010).

## Prefrontal Microcircuit Organization

In order to understand the organization of spatial and object information in the PFC, it is necessary to delve into the representation of stimulus properties at the scale of cortical micro-circuits. Anatomical evidence reveals a regular pattern of axonal terminations into prefrontal neurons, originating either from within the PFC or from association cortices, forming repeating, interdigitated stripes (Kritzer and Goldman-Rakic, 1995; Pucak et al., 1996). More recent studies also reveal a systematic relationship of correlated spiking as a function of distance between neurons and of their spatial tuning, which also points to a regular organization of synaptic inputs (Leavitt et al., 2013). The principles of anatomical input organization with respect to their functional content remain elusive, though the available evidence raises some possible alternatives.

It is well understood that the PFC is not organized in a retinotopic fashion, with only a coarse bias towards peripheral locations in dorsal subdivisions and foveal locations in ventral subdivisions (Suzuki and Azuma, 1983). The lack of a retinotopic organization across the surface of the PFC should not be surprising given that prefrontal neurons are modulated by a wide array of sensory and cognitive factors. Theoretical studies have formally demonstrated the advantage of schemes that contain multiple representations of the same sensory stimulus, mapped to multiple outcomes but each modulated in a different fashion by contextual factors (Salinas, 2004; Rigotti et al., 2013). The lack of a retinotopic map has been readily demonstrated in studies that sampled systematically the surface of the dorsolateral PFC. The same retinal location was found to be represented at multiple electrode tracks, scattered across the surface of cortex (Constantinidis et al., 2001; Meyer et al., 2011). More recent studies, using chronic implants with a regular grid of electrodes to sample a small area of the PFC, also found no obvious organization; neurons representing any spatial location of the contralateral hemifield are observed within the ~3 × 3 mm surface of such an array with electrodes spaced at 0.4 mm apart (Kiani et al., 2015; Bullock et al., 2017). Wider grids of electrodes spaced at 1.5 mm of each other, revealed no obvious pattern of retinal location represented at this larger spatial scale, either (Markowitz et al., 2015).

Even though the surface of the PFC does not correspond to a topographic map of visual space, organization of receptive fields is not random. Evidence suggests a systematic organization of spatial information, at a finer scale, particularly in the posterior dorsal PFC. Simultaneous recordings from closely spaced electrodes have indicated that neurons in proximity of each other (laterally separated by 0.2–0.3 mm) most often represent adjacent spatial locations (Constantinidis et al., 2001). Clustering of neurons with preference for similar spatial locations and similar motion direction tuning was also detected for prefrontal neurons located up to 0.7 mm apart from each other (Masse et al., 2017), as well as in the grid recordings mentioned above Markowitz et al., 2015;Bullock et al. (2017). Anatomical reconstruction of neurons recorded in neurophysiological experiments as electrodes descent into the principal sulcus also indicate a regular transition of memory field location in adjacent micro-columns (Arnsten, 2013). These results raise the possibility that the entire visual hemifield is represented in repeating cortical modules corresponding to the (0.2–0.8 mm) dimensions of the stripe-like zones of axonal terminations (Levitt et al., 1993; Kritzer and Goldman-Rakic, 1995; Pucak et al., 1996).

## Conclusions and Unresolved Questions

A number of conclusions can be drawn from the results that were reviewed here. Anatomical inputs to the PFC are relatively segregated along a dorsal-ventral axis and modulated along an anterior-posterior axis. Functional specialization reflects this anatomical organization so that spatial information is represented predominantly (though not exclusively) in the dorsal PFC. This dominance of spatial information in the dorsal PFC is unaltered by training in working memory tasks, whether they require spatial or object working memory, or combination of both. However, this specialization is highly dependent on: (a) location across the anterior-posterior axis, as higher stimulus selectivity was observed more posteriorly in the dorsolateral PFC and greater dependence to task demands, more anteriorly; (b) time course of responses, with greater sensitivity to spatial location appearing earlier in trials; and (c) representation of peripheral as opposed to perifoveal stimuli, with the former being more distinguishing between dorsal and ventral PFC.

Object working memory has been more difficult to localize with neurophysiological methods. Dorsal prefrontal neurons have broad selectivity for shape and color, and more dorsal prefrontal neurons respond to any given object. On the other hand, the ventral PFC appears better suited for the representation of highly specialized objects like faces. Object selectivity also declines towards more anterior areas and is time-dependent.

A number of unanswered questions remain. One category of questions has to do with the organization of the PFC at the scale of cortical areas and the scale of cortical columns. Is there a functional map of some sort across the surface of prefrontal areas? If so, which are the functional variables that are mapped systematically? What is the role of a prefrontal column within such a map? A combination of fMRI, optical imaging and neurophysiological recordings in non-human primates has made progress in revealing the functional organization of other cortical areas and will likely be instrumental for addressing this question in the PFC.

A second series of questions has to do with the functional implications of activity in different areas of the PFC. The functional role of the different divisions of the PFC will be definitively revealed when a direct link between neuronal activity and behavior can be established. In this context, the question that needs to be addressed is which areas and neurons determine behavior in different working memory and other cognitive tasks? Modern methods of neuronal inactivation and excitation, including through optogenetic manipulation combined with large scale recordings that allow prediction of trial-to-trial deviations of behavior depending on neuronal activity offer promise in answering these questions definitively.

## Author Contributions

CC and X-LQ conceptualized this article, reviewed the literature and composed the text.

## Conflict of Interest Statement

The authors declare that the research was conducted in the absence of any commercial or financial relationships that could be construed as a potential conflict of interest. The reviewer SJC and handling Editor declared their shared affiliation.
